# Hair Dehydroepiandrosterone Sulfate (DHEA(S)) and Cortisol/DHEA(S) Ratio as Long-Lasting Biomarkers of Clinical Syndromes Exhibited by Piglets Early in Life

**DOI:** 10.3390/ani15071032

**Published:** 2025-04-03

**Authors:** Annalisa Scollo, Alessio Cotticelli, Tanja Peric, Alice Perrucci, Alberto Prandi, Paolo Ferrari

**Affiliations:** 1 Department of Veterinary Sciences, University of Torino, 10095 Grugliasco, Italy; annalisa.scollo@unito.it (A.S.); alice.perrucci@unito.it (A.P.); 2 Department of Veterinary Medicine and Animal Production, University of Napoli Federico II, 80137 Napoli, Italy; 3 Department of Agricultural Food, Environmental and Animal Science (DI4A), University of Udine, 33100 Udine, Italy; tanja.peric@uniud.it (T.P.); alberto.prandi@uniud.it (A.P.); 4 CRPA Research Centre for Animal Production, 42121 Reggio Emilia, Italy; p.ferrari@crpa.it

**Keywords:** hair, pig, cortisol, dehydroepiandrosterone sulfate, long-lasting biomarkers, clinical syndromes, allostatic load

## Abstract

Poor health and increased susceptibility to infectious diseases are among the main sources of economic losses in the pig industry worldwide, and they also serve as indicators of compromised animal welfare. However, there is limited information on long-lasting biomarkers of poor health and common infections experienced by piglets early in life. Hair cortisol, dehydroepiandrosterone sulfate (DHEA(S)), and their ratio have been proposed as components of the mammalian stress response due to the activation of the hypothalamus–pituitary–adrenal axis and were investigated in this study using 30 batches of pigs from 16 farms. The research hypothesis was that batches of piglets experiencing clinical syndromes (as indicated by enteric, neurological, cutaneous, and locomotor scores) during suckling would exhibit a different pattern of resilience and allostatic load later in life compared to healthy ones. Hair from 25 gilts per batch were collected at either 3.5 or 9 months of age, and hormone extraction was subsequently performed. The farm of origin and the age of the animals significantly influenced hormone concentrations. Moreover, batches affected by enteric disease showed lower DHEA(S) levels (*p* < 0.0001; 15.89 vs. 23.51 pg/mg) and higher cortisol/DHEA(S) ratio (*p* < 0.0001; 82.83 vs. 55.02) than healthy batches. Similar results were observed in batches with a neurological syndrome (DHEA(S): *p* < 0.0001; 12.91 vs. 19.43; cortisol/DHEA(S) ratio: *p* < 0.0001; 97.15 vs. 70.26 pg/mg). These results suggest that pig hair biomarkers carry an intrinsic and temporally stable signal related to early life health status.

## Simple Summary

The association between early life productive indicators, management practices and subsequent growth performance and survival rate are well known in the pig sector. Nevertheless, there is limited information in the scientific literature on long-lasting biomarkers of poor health and common infections exhibited by piglets early in life. The study aimed to investigate the effect of some common clinical syndromes in suckling piglets on hair cortisol, dehydroepiandrosterone sulfate, and their ratio later in life, as these hormones are suggested in other species to be components of the mammalian stress response, due to the activation of the hypothalamus–pituitary–adrenal axis in the effort to restore homeostatic conditions. Results showed that the farm of origin and the age of the animals influenced hormone concentrations beyond the effect of the clinical syndromes. However, animals experiencing enteric and neurological clinical syndromes early in life showed altered hormonal patterns in their hair several months later. Further studies are needed to investigate the potential of these biomarkers to predict mortality or morbidity in pigs, as already observed in humans suffering from several diseases.

## 1. Introduction

Poor health and increased susceptibility to infectious diseases are among the main sources of economic loss in pig industries worldwide [1], and they are also indicators of animals experiencing compromised welfare [2,3]. Several studies have reported associations between early life productive indicators (e.g., low birth and weaning weight), management practices (e.g., cross–fostering during), and subsequent poor growth performance [4,5,6,7,8,9] and survival rates [10,11,12,13,14]. However, most studies have focused either on herd level factors [3,15,16], specific production stages [6,12,14], or the effects of sow and piglet characteristics and management practices during suckling in relation to poor health and reduced welfare in grower–finisher pigs [17].

Nevertheless, there is limited information in the scientific literature on long-lasting biomarkers of poor health and common infections experienced by piglets early in life. Cortisol, dehydroepiandrosterone (sulfate) (DHEA(S)), and their ratio have been proposed by several authors [18,19,20,21,22,23] as biomarkers influenced by chronic stress, nutritional behaviours, physical activity, drug use, sleep disturbances, psychiatric and neurological disorders, cancer, and other complex pathologies in both humans and animals [24]. However, definitive conclusions regarding the effects of these factors remain elusive, due to the still limited number of studies conducted on humans [25] and, as highlighted in a recent review by Gabai et al. [24], on animals. Cortisol and DHEA(S) are both components of the mammalian stress response, regulated by the activation of the hypothalamus–pituitary–adrenal (HPA) axis, which aims to restore homeostatic balance. The resulting increase in circulating cortisol (a glucocorticoid) following HPA axis activation, in response to rising energetic demands, is defined as allostatic load [26]. While glucocorticoids provide valuable information about HPA axis activity, relying solely on their measurement presents limitations [27,28,29]. Therefore, recent research on human subjects has incorporated DHEA(S)—a “glucocorticoid antagonist”—as an additional biomarker of the function of the axis [30]. Cortisol, DHEA(S), and their ratio are also closely associated with the concept of resilience, defined as the ability of an individual to remain minimally affected by a disturbance or to quickly return to a pre-stressor condition [31]. In swine, these biomarkers have been investigated by only few authors [32,33,34,35]; however, to the best of our knowledge, no studies have explored the release of these steroids in pigs with prior inflammatory or infectious conditions, nor their long-term consequences.

The aim of the present study was to investigate the effect of common clinical syndromes exhibited by suckling piglets (as assessed through enteric, neurological, cutaneous, and locomotor scores) on hair cortisol, DHEA(S), and their ratio later in life. The research hypothesis was that batches of piglets exhibiting clinical syndromes during suckling would display different patterns of resilience and allostatic load later in life compared to healthy ones.

## 2. Materials and Methods

The study was conducted within the framework of the project “Healthy Livestock, Tackling Antimicrobial Resistance through Improved Livestock Health and Welfare”, funded by the European Union’s Horizon 2020 research and innovation program. Ethical review and approval were waived for this study, as it was carried out under field conditions during routine animal management and procedures, in compliance with EU Directive 2008/120 laying down minimum standards for the protection of pigs [36].

The study involved 30 batches of pigs reared in compliance with the requirements of Protected Designation of Origin (PDO) ham consortia. It lasted two years and covered all seasons. The batches came from 16 farms: 4 weaning sites (approximately from 7 to 35 kg of live weight, i.e., up to 3.5 months of age; 8 batches) and 12 finishing sites (approximately from 35 kg to a slaughter weight of 170 kg, i.e., up to 9 months of age; 22 batches) randomly selected through convenience sampling from a list provided by a contractor under a contract farming agreement. All farms raised pigs in environmentally controlled buildings with partially slatted floors. Convenience sampling ensured that all pigs enrolled in the study originated from the same farrowing site, which hosted 1200 sows with identical genetics (Danish Genetics—Danish hybrid^®^ × Topigs Norsvin—Talent^®^, ’s-Hertogenbosch, The Netherlands) and early-life management. The average number of weaned piglets per sow per year was 30.6, with an average of 16.3 live-born piglets per farrowing and a stillbirth rate of 5.1%. Sows and litters were housed in conventional farrowing crates with slatted flooring and a solid resting area under the piglet nest. The commercial farrowing site operated on a 3-week batch system [37], producing around 2000 piglets per batch. Sows were vaccinated against porcine reproductive and respiratory syndrome virus (PRRSV), porcine circovirus 2 (PCV2), parvovirus, *Erysipelothrix rhusiopathiae*, and *Mycoplasma hyopneumoniae*. Piglets were vaccinated against *Mycoplasma hyopneumoniae*, PCV2, PRRSV, and *Escherichia coli* F4/F18. In addition, they were surgically castrated and tail-docked at 3 days of age, receiving intramuscular meloxicam 5 mg/mL for pain relief along with an iron injection. Antibiotics were administered individually and only in case of disease, following veterinary recommendations.

**Figure 1:**
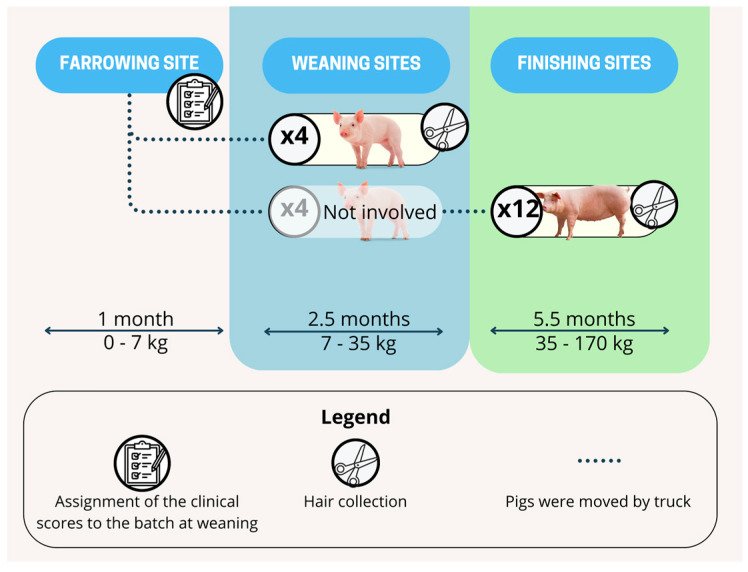
Timeline of the study. Every three weeks, a batch of piglets was transported by truck from the farrowing site to one of the four weaning sites involved in the study. At 3.5 months of age, they were again transported by truck to one of the 12 finishing sites, where they remained until slaughter. A total of 8 batches were monitored at the weaning sites and 22 at the finishing sites, using 200 and 550 individual hair samples, respectively.

Every three weeks, after a 28-day suckling period, a batch of piglets was weaned at the farrowing site and transported by truck to one of the weaning sites involved in the study. At 3.5 months of age, they were transferred to one of the finishing sites, where they remained until slaughter. If the finishing site had a smaller capacity than the corresponding weaning site, surplus pigs were moved to other locations and excluded from the study. Conversely, if the finishing site had a larger capacity, additional pigs from the same farrowing site (three weeks older), physically separated from the original group, were added but not included in the study (Figure 1). All farms were located in northern Italy, where heavy pigs are primarily reared for PDO ham production. This area accounts for 80% of national pig production [38]. All farms adhered to rearing conditions compliant with EU Directive 120/2008 laying down minimum standards for the protection of pigs.

### 2.1. Clinical Scores

During the suckling period (average duration: 27 days), a technician trained by an experienced swine veterinarian recorded the number of piglets showing clinical signs attributable to four clinical syndromes, historically common and recurrent on the farm: enteric, neurological, cutaneous, and locomotor. Clinically affected piglets were ear-tagged using four different colours, one per each syndrome. Although the entire study was based only on clinical observations, the main infectious agents responsible for the diseases were investigated during the study and are summarised in Table 1. Laboratory analyses were performed on at least three clinically affected live piglets per batch (for enteric, cutaneous, and locomotor syndromes), or on euthanized/freshly dead piglets (for the neurological syndrome). All piglets selected for laboratory analysis had not been treated with antibiotics and were sampled during the suckling period as soon as clinical signs appeared.

On the day of weaning, a researcher classified each batch as either healthy (score 0) or clinically affected (score 1) for each syndrome, based on the prevalence of clinically affected piglets recorded during suckling. The thresholds used to classify a batch as clinically affected (Table 1) correspond to the first quartile of the distribution of cases observed for each syndrome during a 12-month data collection period prior to the study. A clinical index was created to identify batches exhibiting more than one clinical syndrome with a score of 1 simultaneously.

When pigs were moved from the farrowing site to the weaning sites, and later to the finishing sites, pen groups were formed based on health status, according to the clinical signs observed in suckling piglets and tracked via ear-tags. Separation between healthy and clinically affected animals by pen was maintained until slaughter.

### 2.2. Hair Sampling and Analysis

Hair samples (approximately 100–150 mg) were collected from 25 randomly selected gilts from each of the 30 batches [40]. In clinically affected batches, gilts were selected from among ear-tagged animals. In the eight weaning batches, hair sampling was performed 10 weeks after the pigs arrived at the weaning facilities (average age: 3.5 months); in the 22 finishing batches, sampling was performed 22 weeks after arrival at the finishing facilities (average age: 9 months). None of the finishing batches had previously been sampled also at the weaning site. Only gilts were sampled in this study, since gender has a significant effect on steroid concentrations, as previously reported by Bergamin et al. [32]. Animals were randomly selected from each farm and, after gentle restraint, hair samples were carefully collected by shaving close to the skin using electric clippers on the dorsal area of the neck, behind the ears. This area was chosen because it is generally cleaner than other parts of the body. The shaved hair samples were placed in paper envelopes, individually labelled, and stored in the dark and at room temperature until analysis. A total of 750 hair samples were collected. At the time of sampling, all pigs had recovered from any possible clinical syndromes previously observed.

Hair strands were washed, extracted, and analysed following the protocols described by Bergamin et al. [32] for hair cortisol and by Fusi et al. [41] for hair DHEA(S) concentrations. Washing with isopropanol is essential to minimise the risk of extracting steroids deposited on the surface of the hair through sweat and sebum.

### 2.3. Statistical Analysis

Data were analysed using XLSTAT 2022.2.1 (Addinson, TX, USA, 2022). Pigs were considered as measures within each batch, which was treated as the statistical unit. Differences in hair cortisol, DHEA(S), and their ratio were assessed using a mixed model, with farm included as a random effect. Multiple and pairwise comparisons between factors (age, clinical score, and their interactions) were performed using Tukey’s honest significant difference (HSD) test for multiple comparisons. A Shapiro–Wilk test was used to verify the normality of the data. Variables that did not follow a normal distribution (cortisol and cortisol/DHEA(S) ratio) were log transformed prior to analysis to correct for their non-normality. Results are presented as mean ± standard deviation. The clinical index was used as a model to investigate intra–score trends of the hormones and their ratio throughout the pigs’ life.

## 3. Results

The average number of pigs reared on the visited farms was 1204 ± 867 (minimum = 300; maximum = 2770), with an average mortality rate of 4.8 ± 1.7% (minimum = 2.7; maximum = 8.8). A total of eight batches from weaning sites and 22 from finishing sites were investigated, through the analysis of 200 and 550 hair samples, respectively. The frequency of each clinical syndrome, as assessed at weaning, was as follows: 23/30 batches (76.7%) for enteric syndrome, 7/30 (23.3%) for neurological syndrome, 13/30 (43.3%) for cutaneous syndrome, and 15/30 (50%) for locomotor syndrome. Batches simultaneously exhibiting more than one syndrome (clinical index = 1) were observed in 25/30 cases (83.3%).

**Figure 2:**
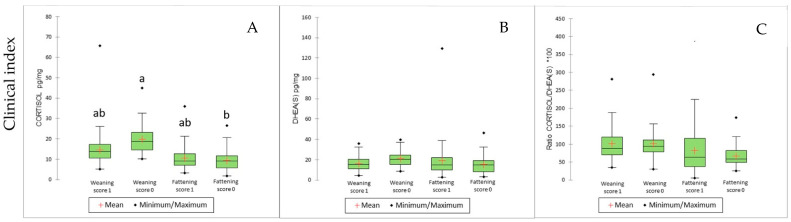
Cortisol (**A**) and DHEA(S) (**B**) concentrations (pg/mg), and cortisol/DHEA(S) ratio (**C**) according to age, in batches classified as healthy (=0) or clinically affected (=1) at an average age of 27 days based on the clinical index. A total of 200 hair samples were collected at the weaning sites and 550 at the finishing sites, each from a different pig. a, b mean differences among ages and scores (weaning, 3.5 months vs. finishing, 9 months) at *p* < 0.05.

Results for cortisol, DHEA(S), and cortisol/DHEA(S) ratio from multiple comparisons across different clinical scores and age groups are reported in Table 2. The effect of the farm was significant for all three hair parameters (*p* < 0.002). Regardless of clinical syndromes, age had a significant effect on cortisol concentration (*p* = 0.005; weaning sites = 14.64 vs. finishing sites = 9.15 pg/mg), as well as on the cortisol/DHEA(S) ratio (*p* = 0.032; weaning sites = 92.32 vs. finishing sites = 60.25). In addition to age, batches scored 1 (clinically affected) for enteric syndrome showed significantly lower DHEA(S) concentrations (*p* < 0.0001; score 1 = 15.89 vs. score 0 = 23.51 pg/mg) and higher cortisol/DHEA(S) ratios (*p* < 0.0001; score 1 = 82.83 vs. score 0 = 55.02) compared to healthy batches (score 0). A similar trend was observed in batches scored 1 for neurological syndrome, with lower DHEA(S) concentrations (*p* < 0.0001; score 1 = 12.91 vs. score 0 = 19.43 pg/mg) and higher cortisol/DHEA(S) ratios (*p* < 0.0001; score 1 = 97.15 vs. score 0 = 70.26). Conversely, batches scored 1 for locomotor syndrome showed higher DHEA(S) concentrations (*p* = 0.047; score 1 = 19.52 vs. score 0 = 17.43 pg/mg) and lower cortisol/DHEA(S) ratios (*p* = 0.011; score 1 = 68.95 vs. score 0 = 77.60) than healthy batches. No significant differences were found between scores 0 and 1 for the cutaneous syndrome or the clinical index. For the latter, the values were as follows: cortisol 12.2 vs. 11.2 pg/mg (*p* = 0.564), DHEA(S) 21.9 vs. 17.2 (*p* = 0.376), and cortisol/DHEA(S) ratio 76.5 vs. 61.4 (*p* = 0.359). However, when the clinical index was analysed as a model for intra-score hormonal trends over time (from 3.5 to 9 months of age), batches scored 0 showed a statistically significant decrease in cortisol concentration (from 18.7 to 8.0 pg/mg; −57.2%; *p* = 0.045), unlike batches scored 1 (from 13.5 to 9.3 pg/mg; −31.1%; *p* > 0.05). A similar trend was observed for the cortisol/DHEA(S) ratio, though without statistical significance: a decrease from 93.0 to 40.1 (−56.9%) in batches scored 0, and from 92.1 to 63.6 (−30.9%) in batches scored 1 (Figure 2).

## 4. Discussion

The aim of the study was to investigate the effect of common clinical syndromes in suckling piglets (as identified by enteric, neurological, cutaneous, and locomotor scores) on hair cortisol, DHEA(S), and their ratio later in life. These biomarkers are indicative of resilience and allostatic load in various animal species. The authors hypothesised that batches of piglets exhibiting clinical syndromes during suckling would display a different endocrine profile later in life compared to healthy ones.

One of the most common infectious causes of poor health during suckling is neonatal diarrhoea, which may be triggered by several pathogens (e.g., *Escherichia coli*, *Clostridium perfringens*, *Clostridioides difficile*, *Cystoisospora suis*, rotavirus, and *Cryptosporidium parvum* [42]). This is followed in frequency by *Streptococcus suis* infections, which are responsible for neurological signs [43]. Other frequent causes of impaired neonatal health, especially in piglets with low immunological protection, include exudative epidermitis and infectious arthritis. The former is a skin infection primarily caused by *Staphylococcus hyicus*, particularly by virulent strains that produce exfoliative toxins [44]. The latter, associated with locomotor disorders, is mainly due to hemolytic streptococci, although staphylococci and *E. coli* are also commonly isolated [45].

To explore the potential effect of these clinical syndromes on cortisol, DHEA(S), and their ratio later in life, hair was selected as the biological matrix for hormone analysis. Hair is unaffected by circadian fluctuations or short-term influences on hormone levels [46], and sampling is non-invasive for the animals. Moreover, it provides a retrospective timeline of hormone exposure [46,47,48], offering an integrated measure of hormone concentrations over medium– and long–term periods [49]. Hormones are deposited in the hair shaft and can reflect levels from birth until the time of sample collection (3.5 or 9 months of age in this study), excluding the final 15 days prior to sampling [50,51].

Before delving into the complex interactions between the analysed factors, it is important to highlight that both the farm of origin and the age of the animals influenced hormone concentrations, independently of the clinical syndromes. This farm effect was expected, as both cortisol and DHEA(S) are secreted following the activation of the HPA axis, which can be triggered by a wide range of stressors, including environmental and management-related factors (e.g., pen size, environmental enrichments, transportation, human interaction), as reviewed by Whitham et al. [52]. Notably, the expression of clinical syndromes itself may also be influenced by specific farm characteristics. To account for this variability in the statistical analysis, a random-effects model was selected, incorporating the farm effect along with other variables under investigation (primarily hormone concentrations). According to Herdt et al. [53], random-effects models are designed to estimate the probability that a specific farm does not belong to the reference population, by applying appropriate computational methods. This approach is particularly recommended in cases such as the present study, where farm means are calculated within a clinical framework and the outcomes are influenced by both individual variability and a multitude of environmental and managerial factors affecting all animals on the farm.

Hair cortisol and the cortisol/DHEA(S) ratio were also affected by age, with statistically significant differences observed between weaning and finishing groups. Hair samples collected at 3.5 months of age showed significantly higher cortisol concentrations and cortisol/DHEA(S) ratios compared to those collected at 9 months. Although several authors have reported the opposite trend in primates—i.e., hypercortisolism and higher cortisol/DHEA(S) ratios as age–related changes in older individuals [54,55,56]—some studies in other mammals have described hormonal patterns consistent with the present findings. For example, among adult male killer whales, those considered “aged” (at least 31 years old) showed lower glucocorticoid concentrations than younger individuals [57]. In livestock, a decrease in cortisol concentrations has been observed in calves, which was hypothesised to be due to a reduction in environmental stressors as the animals moved further away from the weaning period [58]. In fact, several studies have demonstrated that high cortisol concentrations are physiological in neonates, given the hormone’s role in modulating multiple systems [59,60]. In the authors’ opinion, and in agreement with Peric et al. [58], the higher cortisol concentrations and cortisol/DHEA(S) ratios observed in the present study among weaned piglets compared to older animals may reflect the high levels of stress experienced during this life stage. Sudden separation from the sow, abrupt dietary changes, modifications in physical and social environments, and transportation are among the most invasive stressors faced by piglets immediately after weaning [61]. Hair samples collected from both weaners and fatteners reflect hormonal variations resulting from postnatal experiences, as well as prenatal and perinatal events [59,60]. However, by 9 months of age, the cumulative hormonal deposition may appear “diluted” across the total hair length, thereby masking earlier peaks in hormone levels. Furthermore, hair growth is intermittent in all species studied so far and occurs in cycles consisting of an active growth phase (anagen), followed by a transitional phase (catagen), and a resting phase (telogen). During anagen, hair produced in previous cycles may be lost. This process of partial hair replacement—observed also in swine [62]—could lead to the loss of early-life hormonal information.

Batches classified with clinical enteric and neurological syndromes (scored at 27 days of age) showed lower DHEA(S) concentrations and higher cortisol/DHEA(S) ratios compared to batches classified as healthy. The effect of these syndromes was strong regardless of age (*p* < 0.0001) and, although not always supported by full statistical significance—likely due to the limited number of batches per age group and the strong farm effect—a numerical difference was still evident at both 3.5 and 9 months of age. This difference emerged even though, at the time of both sampling points (weaning and finishing), the clinical syndromes observed at 27 days had resolved, and samples were taken from clinically healthy pigs. Although DHEA(S) has been characterised as a glucocorticoid antagonist, an immunostimulant, a biomarker of ageing, and a neuroprotective hormone, and may increase in response to acute stress with a protective function [63,64,65], chronic stress can dysregulate the HPA axis and result in decreased DHEA(S) secretion [25,30]. As noted by Straub et al. [66], research in humans suggests that both acute and chronic stress or disease can affect plasma DHEA(S) concentrations, albeit in different ways. Indeed, reductions in this hormone have been reported in chronic inflammatory conditions and diseases, such as mood disorders, chronic pain syndromes (e.g., fibromyalgia), and inflammatory bowel disease [66]. In particular, and in agreement with the findings on enterically affected pigs in the present study, Sugaya et al. [67] found that chronically reduced DHEA(S) concentrations in humans were associated with the severity and duration of abdominal pain, diarrhoea, and infrequent bowel movements. The authors speculated that chronically low levels of DHEA(S) may not be a consequence but rather a cause of gastrointestinal symptoms, due to a reduced immunomodulatory effect of DHEA(S), in contrast to the immunosuppressive effects of cortisol. This hypothesis supports the need for further investigation into a potential link between DHEA(S) and foetal stress in piglets during late gestation and at birth, which may act as a trigger for enteric disorders. Although studies on DHEA(S) in diseased pigs are limited, the results of the present study align with those of Schurr et al. [35], who reported DHEA(S) concentrations below baseline levels following experimental administration of *Escherichia coli* lipopolysaccharides. Regarding the lower DHEA(S) concentrations observed in batches classified with a neurological syndrome, several authors have reported that this hormone and its metabolites are altered in various neurodegenerative conditions. For instance, Sosvorova et al. [68] found decreased concentrations of DHEA metabolites in the cerebrospinal fluid of human patients with hydrocephalus compared to controls, suggesting that such changes may reflect inflammatory processes in the brain. In the present study, neurological classification was based on the isolation of *Streptococcus suis* from the brain samples, a pathogen known to cause severe systemic and cerebral inflammatory responses due to meningitis in pigs [69].

Given the antagonistic relationship between cortisol and DHEA(S), assessing both hormones simultaneously may provide a more comprehensive picture of glucocorticoid activity, which is often expressed through their ratio [25]. The findings of the present study are consistent with previous literature, where decreased DHEA(S) concentrations are typically associated with increased cortisol/DHEA(S) ratios (see the review written by Whitham et al. [52]).

In contrast to other findings, batches displaying cutaneous disorders at 27 days of age showed higher DHEA(S) concentrations and lower cortisol/DHEA(S) ratios compared to healthy animals at 3.5 months of age. In this case, DHEA(S) appears to follow the pattern typically observed in response to acute stressors [24]. Indeed, the greasy pig disease used to classify a batch as clinically affected in the present study is described as a skin infection that often occurs acutely in pigs aged 3–10 weeks [69,70], usually followed by recovery or death. It is possible that the hormonal biomarkers used in this study are not able to retain information about cutaneous disorders over time, up to 9 months of age. Alternatively, this information may have been lost due to the death (unmonitored in this study) of affected animals, considering the frequently high mortality rate associated with this condition [44]. Although some studies have confirmed the beneficial effects of DHEA(S) administration on atopic dermatitis–like skin lesions and skin ageing in mice and humans [71,72], others have failed to find a correlation between skin diseases and circulating androgen concentrations in girls [73]. Given the complete absence of such research in pigs, further studies are strongly encouraged.

DHEA(S) concentrations and cortisol/DHEA(S) ratios also differed in pigs showing a locomotor syndrome at 27 days of age compared to healthy pigs when sampled at 9 months. The literature on DHEA(S) release in lame animals is limited (mostly involving dairy cows [74,75,76]), and the most recent review on the topic [24] highlighted that current evidence is inconclusive and difficult to compare. O’Driscoll et al. [74] found results similar to those in the present study, including in lame cows with sole ulcers. However, in the authors’ opinion, an association between locomotor disorders at 27 days of age and hormone levels at 9 months should be interpreted with caution, especially since no comparable association was observed at the intermediate sampling point (3.5 months). One possible explanation is that the hormones analysed in this study may not be optimal biomarkers for locomotor disorders, as previously suggested by other authors [75,76,77]. Alternatively, piglets showing locomotor issues early in life may be predisposed to developing other disorders later on, such as altered joint vascularisation or osteochondrosis, which are relatively common in modern growing pigs [78]. More studies are needed to explore DHEA(S) release in lame pigs, including investigations into the aetiology, duration, and inflammatory nature (acute or chronic) of the condition, as well as associated pain levels.

In this study, the creation of a clinical index incorporating multiple syndromes failed to identify significant differences between the most severely affected batches (at least two syndromes) and those exhibiting only one syndrome. Despite this, the average hair cortisol concentration observed in finishing batches falls within the range previously reported in the recent exploratory study on pig hair cortisol conducted on 20 finishing farms at 165 ± 10 days of age (7.99–9.39 vs. 4.8–54.5 pg/mg [40]). However, when intra-score comparisons were performed between 3.5 and 9 months using the clinical index, cortisol concentrations (and the cortisol/DHEA(S) ratio, though not statistically significant) showed a marked decrease in batches scored 0 (−57.2 and −56.9%, respectively), whereas the decrease was less pronounced in batches scored 1 (−31.1 and −30.9%). This could indicate a HPA axis response caused by prolonged pathogen exposure or disease history in affected batches. As such, batches scored 1 may find themselves in a “disadvantaged” physiological state compared to healthy ones, still needing to restore endocrine homeostasis. Moreover, although not statistically significant, the observation of the boxplots in Figure 2 may suggest that pigs affected by more than one syndrome (clinical index = 1) display a depressed HPA axis activity (lower cortisol and DHEA(S) concentrations) compared to healthier ones. A similar pattern was previously observed in healthy versus sick foals [79].

Just as subclinical cases require a specific diagnostic approach, the same consideration applies to alterations in DHEA(S) and the cortisol/DHEA(S) ratio resulting from diseases experienced earlier in life: diagnosis cannot be based solely on hormone concentrations measured in hair [80]. Indeed, the pathogen may no longer be present at the time of sampling, and the observed hormone levels cannot be considered pathognomonic for any specific disease. At the same time, it is important to bear in mind that treatment efficacy may be compromised in animals experiencing high allostatic load. Nonetheless, further research in livestock may support the hypothesis—already proposed in human medicine for several diseases (e.g., Alzheimer’s disease and metabolic syndrome)—that altered hormone concentrations may serve as predictive biomarkers for mortality or morbidity [81].

The results of the present study should be interpreted in light of some limitations. First, endocrine data refer exclusively to female pigs. Additionally, although a random-effects model was applied, weaning and finishing animals came from different farms and were sampled at different ages, which may have introduced some bias. For example, a delayed resolution of clinical syndromes or unmonitored stressful events may have occurred between weaning and 9 months of age. More generally, the potential impact of eustress cannot be excluded and may have influenced the results, thus warranting further investigation. Finally, the exclusive reliance on clinical observation for health classification did not allow for the assessment of the incidence of specific pathogens or subclinical conditions that may have affected the findings.

## 5. Conclusions

The results obtained in this study are promising as they suggest that DHEA(S) concentrations and the cortisol/DHEA(S) ratio in pig hair may carry an intrinsic biological signal. The altered hormonal profiles observed in clinically healthy animals could reflect the allostatic load experienced by farmed pigs affected by common diseases, potentially indicating reduced welfare and production efficiency.

## Figures and Tables

**Table 1:** Description of the investigations performed for each clinical syndrome observed pre-weaning, and the thresholds used to classify a batch as clinically affected (score 1).

Clinical Syndrome	Laboratory Analyses and Diagnosis	Threshold to Classify the Batch as Clinically Affected (Score 1)
Enteric	Bacteriology on rectal samples isolated *Escherichia coli*. Colonies surrounded by a zone of lysis after overnight growth at 37 °C on blood agar were classified as haemolytic, and detection of virulence factor genes F18, STa, and STb was obtained by PCR	More than 10% of piglets with profuse yellowish diarrhoea
Neurological	Bacteriological analysis of brain samples (cultured on blood agar supplemented with NAD and Gassner agar) led to the isolation of *Streptococcus suis*	More than 0.6% of piglets with neurological signs
Cutaneous	Bacteriology from skin wounds or pus (cultured on MacConkey agar) allowed to isolate *Staphylococcus* spp. The identification of *S. hyicus* was obtained by PCR (diagnosis: exudative epidermitis or “Greasy pig disease” [39])	More than 5% of piglets with exudative epidermitis
Locomotor	Bacteriology from intra–articular fluid (cultured on MacConkey agar) allowed to isolate *Staphylococcus* spp. The identification of *S. hyicus* was obtained by PCR	More than 1.5% of piglets with at least one leg with signs of inflammation without traumas.
Clinical Index	–	Presence of more than one clinical syndrome scored 1

**Table 2:** LS means ± standard deviation of cortisol and DHEA(S) concentrations (measured at the weaning or finishing site), and their ratio, for each clinical score assigned to the pigs at an average age of 27 days (healthy = 0; clinically affected = 1). A total of 200 hair samples were collected at the weaning sites and 550 at the finishing sites, each from a different pig.

Factors		Cortisol, pg/mg		DHEA(S), pg/mg		Cortisol/DHEA(S) Ratio × 100	
		Weaning	Finishing	Weaning	Finishing	Weaning	Finishing
Enteric score	0	17.18 ± 1.05	8.28 ± 1.05	23.15 ± 1.54 *	23.88 ± 1.34	73.73 ± 1.08	41.07 ± 1.07 *
	1	13.30 ± 1.04	9.33 ± 1.02	13.84 ± 1.16 *	17.93 ± 0.64	105.62 ± 1.05	64.96 ± 1.03 *
Neurological score	0	14.68 ± 1.03	9.05 ± 1.02	18.41 ± 1.01	20.44 ± 0.68	86.57 ± 1.05 **	57.03 ± 1.03
	1	14.35 ± 1.08	9.39 ± 1.04	10.47 ± 2.43	15.35 ± 1.09	138.53 ± 1.13 **	68.14 ± 1.05
Cutaneous score	0	14.69 ± 1.04	9.23 ± 1.03	15.83 ± 1.07 *	22.13 ± 0.82	102.13 ± 1.05 ***	52.06 ± 1.04
	1	14.47 ± 1.06	9.08 ± 1.03	22.11 ± 1.88 *	16.24 ± 0.82	66.89 ± 1.10 ***	67.85 ± 1.04
Locomotor score	0	15.03 ± 1.05	9.34 ± 1.03	18.34 ± 1.48	16.52 ± 0.78 *	87.87 ± 1.08	68.54 ± 1.04 **
	1	14.40 ± 1.04	8.83 ± 1.03	16.69 ± 1.19	22.35 ± 0.86 *	95.22 ± 1.06	49.92 ± 1.04 **
Clinical index	0	18.67 ± 1.06	7.99 ± 1.02	21.27 ± 1.89	22.64 ± 1.53	92.96 ± 1.10	40.52 ± 1.08
	1	13.51 ± 1.04	9.33 ± 1.02	16.02 ± 1.06	18.41 ± 0.62	92.09 ± 1.05	63.62 ± 1.03

* *p*-value < 0.05; ** *p*-value < 0.01; *** *p*-value < 0.001.

## Data Availability

The original contributions presented in this study are included in the article. Further inquiries can be directed to the corresponding author.
